# Health Risks Associated with Adopting New-Generation Disposable Products Among Young Adults Who Use E-Cigarettes

**DOI:** 10.3390/ijerph21101375

**Published:** 2024-10-18

**Authors:** Shuyao Ran, James J. Yang, Megan E. Piper, Hsien-Chang Lin, Anne Buu

**Affiliations:** 1 Department of Management, Policy, & Community Health, University of Texas Health Science Center, 1200 Pressler Street, Houston, TX 77030, USA; shuyao.ran@uth.tmc.edu; 2 Department of Biostatistics and Data Science, University of Texas Health Science Center, 1200 Pressler Street, Houston, TX 77030, USA; james.j.yang@uth.tmc.edu; 3 Center for Tobacco Research & Intervention, Department of Medicine, School of Medicine and Public Health, University of Wisconsin, 1930 Monroe Street, Suite 200, Madison, WI 53711, USA; mep@ctri.wisc.edu; 4 Department of Applied Health Science, School of Public Health, Indiana University-Bloomington, 1025 E. 7th Street, SPH 116, Bloomington, IN 47405, USA; linhsi@indiana.edu; 5 Department of Health Promotion and Behavioral Sciences, University of Texas Health Science Center, 7000 Fannin Street, Houston, TX 77030, USA

**Keywords:** electronic cigarette, disposable product, e-cigarette dependence, combustible tobacco, respiratory symptom

## Abstract

New-generation disposable e-cigarettes have become increasingly popular among young adults in the USA since the FDA’s partial flavor ban. This study aims to examine longitudinal changes in health risks among young adults who adopted these novel products, as well as the health effects of device types beyond the effects of other important e-cigarette characteristics. This study recruited e-cigarette users via voluntary response sampling from three college campuses in the USA to respond to four-wave online surveys conducted in four consecutive semesters. Among the participants who adopted disposables during the study, their health risks (dependence symptoms, respiratory symptoms, combustible tobacco use) and e-cigarette consumption characteristics (use frequency, nicotine concentration and flavors) before and after the adoption were compared using paired-sample t- or McNemar’s tests. Generalized linear mixed models with a random intercept were conducted on data from the entire sample to investigate the effects of device type (tank, cartridge/pod, disposable) on health risks, controlling for other e-cigarette consumption characteristics. The study sample of 650 e-cigarette users were, on average, 20 years old, with 49% being male, 70% being White, and 13% being Hispanic. Adopting disposables may increase secondary dependence motives (t = 2.42, *p* < 0.05) and the use of higher levels of nicotine concentration (t = 2.09, *p* < 0.05) and sweet flavors (x^2^ = 22.53, *p* < 0.05) but decrease the number of times of vaping per day (t = −2.18, *p* < 0.05) and the use of menthol flavors (x^2^ = 4.57, *p* < 0.05). Tank use is associated with a higher level of primary dependence motives (b = 0.1998, *p* < 0.05) and a greater odds of using combustible tobacco (b = 0.4772, *p* < 0.05). Although disposable use is not associated with the likelihood of using combustible tobacco, it is associated with higher levels of both primary (b = 0.2158, *p* < 0.05) and secondary (b = 0.2533, *p* < 0.05) dependence motives. It is not the device type, but rather the frequency of vaping, that affects respiratory symptoms (b = 0.0602, *p* < 0.05). The findings indicate that when young adults switch to disposables, their e-cigarette dependence and use of sweet-flavored e-liquids increase. Even after controlling for use frequency, nicotine concentration and flavors, using disposables is related to not only instrumental motives that are influenced by psychological and environmental contexts but also heavy, automatic use that can operate without environmental cues. Given the health risks associated with disposable e-cigarettes, more comprehensive tobacco product regulations that consider the impact of device types may be needed.

## 1. Introduction

The US Food and Drug Administration (FDA) implemented the partial flavor ban on closed-cartridge rechargeable electronic cigarettes (e-cigarettes) with flavors other than tobacco and menthol on 6 February 2020. Since then, the popularity of a new generation of pre-charged and pre-filled disposable e-cigarette products with a variety of flavors (e.g., Puff Bars) has grown exponentially [1]. A recent study analyzing national survey data from a longitudinal cohort of youth in the US found that a large proportion of cartridge users in 2019 switched to disposable users in 2021 [2]. Another survey study of adolescents and young adults also showed that the baseline use of disposable (versus only non-disposable) devices was associated with higher odds of continued e-cigarette use and a higher frequency of use 8 months later [3]. The growth in the disposable e-cigarette market, which accounts for 92.8% of 6287 e-cigarette products sold in the US during the 6-month period ending on 16 June 2024 [4], has been driven by products that hold more e-liquid, e-liquid with a higher nicotine concentration, and declining prices [5]. Disposable e-cigarettes also come in sweet flavors and bright colors that are particularly attractive to young people [6]. Thus, evaluating the health risk associated with these highly popular products, relative to the risk with earlier-generation products (e.g., tank, cartridge, pod), will have important policy implications.

Few studies have investigated the risks of e-cigarette dependence, the use of combustible tobacco, and the pulmonary effects associated with disposable e-cigarettes. A study of young adults who used disposable e-cigarettes found that a greater number of days using e-cigarettes was cross-sectionally associated with using ice-flavored (vs. fruit/sweet-flavored) e-cigarettes and reporting any vaping dependence symptoms [7]. Conversely, a recent study of youth and young adults who used e-cigarettes showed that more tank users endorsed two e-cigarette dependence symptoms—reaching for a device without thinking about it and vaping more before going into a situation where vaping is not allowed—compared to those who used pod or disposable e-cigarettes [8]. A meta-analysis using cross-sectional data from four samples of youth and young adults who used e-cigarettes showed that frequently using disposable devices (vs. pods) and multiple devices (vs. pods) was associated with greater odds of combustible tobacco use [9]. Another meta-analysis using the same data found that although a greater frequency of e-cigarette use was associated with bronchitic symptoms, such associations did not differ by device type [10]. However, that study aggregated the earlier-generation disposable e-cigarettes (cigalikes) and the new-generation disposable e-cigarettes (e.g., Puff Bars) together as the same type, despite their significantly different biological effects on human airways [11]. In summary, while the extant literature has found a cross-sectional association of disposable e-cigarette use with dependence symptoms, combustible tobacco use, and respiratory symptoms, the literature is sparse and inconsistent.

This study uses the four-wave longitudinal data collected from a sample of college students who used e-cigarettes during the period of rapid growth of disposable e-cigarette products that particularly targeted young people (from late 2019 to early 2023) to fill critical knowledge gaps of the literature. The research objectives include the following: (1) identifying a group of participants who adopted the new disposable products during the study period and conducting within-person comparisons to examine the changes in health risks, including e-cigarette dependence symptoms, respiratory symptoms, and concurrent use of combustible tobacco, as well as changes in e-cigarette consumption characteristics such as use frequency, nicotine concentration, and flavors; and (2) investigating the effects of e-cigarette devices (tank, cartridge/pod, disposable) on health risks, adjusting for the effects of demographic background and other e-cigarette consumption characteristics.

## 2. Methods

### 2.1. Study Sample Recruitment

This study recruited 686 participants from three college campuses in the midwestern and southern United States from fall 2019 to fall 2021 by distributing study flyers to students through email listservs, social media, online classified advertising sites, bulletin boards, tabling events, and brief presentations in undergraduate classes (i.e., voluntary response sampling). The eligibility criteria were (1) e-cigarette use at least once per week in the last 4 weeks; (2) no intention to quit e-cigarette use in the next 30 days; (3) first- to third-year undergraduates; (4) ownership of a smartphone; and (5) using a tank, cartridge, pod, or new-generation disposable e-cigarette.

### 2.2. Study Design & Procedure

This longitudinal study assessed participants’ e-cigarette and other tobacco use using a web survey and 7-day ecological momentary assessment (EMA) via a smartphone app in each of four consecutive semesters. The study protocol was approved by the Institutional Review Board (IRB) of the University of Texas Health Science Center at Houston (HSC-SPH-19-0391). During the baseline assessment, participants provided informed consent, completed assessments of demographic background and lifetime substance use, and received training on the EMA protocol and a payment of USD 20. Participants were also offered the opportunity to earn an extra USD 20 by providing a saliva cotinine sample.

After the baseline assessment, an email was sent to the participant with a link to web survey that measured e-cigarette consumption and related health outcomes. The survey also asked about the student’s typical wake-up time and sleep time on weekdays and weekends to determine the individualized period of computer-initiated prompts during the 7-day EMA data collection. Participants were compensated USD 20 for each survey they completed. The participant then initiated the EMA data collection period (see detailed information in [12]). The same procedure of administering the web survey and then the EMA protocol was repeated for each of the following three semesters. Web survey data from 650 participants whose device types could be accurately coded at one or more waves were included in the analyses to address the research questions and examine the health risks associated with the types of e-cigarettes.

### 2.3. Measures

**Procedure for coding e-cigarette device types.** Each semester, the web survey asked participants to identify the device types they currently used from tank, cartridge, or pod (check all that apply). If they checked cartridge or pod, they were asked to specify the brand(s) used in past 30 days by choosing from seven popular brands (e.g., JUUL, Vuse) and/or filling out the brand/product name(s). We created a binary code for the cartridge/pod and a binary code for the disposable based on participant responses. For the brands that only made reusable devices (e.g., JUUL) or those that only made disposables (e.g., Puff Bar), the coding procedure was straightforward. For those brands currently offering both reusable and disposable products, the Google Trends data were employed to identify the approximate time when a brand started to make disposable products available. We used the term “disposable” and the specific disposable product names following the brand name to download the corresponding US-level, Google Trends data containing the relative search volume (RSV), which was normalized between 0 and 100, from September 2019 to May 2023 (our data collection period). For the brands with minimal monthly RSVs (<10) during the study period (e.g., Vuse), the disposable variable was coded as “0” and the cartridge/pod variable was coded as “1”, because the likelihood that our participants would use disposables produced by the brand was very low. This coding also applied to those brands that had slightly higher RSVs overall (e.g., SMOK), until we observed a spurred increase in monthly RSV (>50). Beyond that point of time, both variables were coded as “missing” due to the high degree of uncertainty. Additionally, when information was insufficient to identify the availability of disposables for any write-in brand names (e.g., UWELL), we assigned missing values to both binary variables. The date of the FDA flavor ban was not used for the coding process as participants’ access to products may not have been affected because they could stock up or buy flavored products from alternative sources [13]. In summary, three binary variables were included in the analysis: the variable for cartridge/pod and the one for disposable were generated by the coding process described above; the variable for tank directly came from participants’ responses to the device type question on the survey. These three binary variables are not mutually exclusive because participants could use multiple devices during the same period of time.

**E-cigarette consumption characteristics.** In addition to the device type, this study investigated the following e-cigarette consumption characteristics that may be associated with health risks: (1) the number of days vaping in the past 30 days (0–30); (2) the number of times vaping per day (on a scale of 1–6; see Table 1 for categorical options); (3) the nicotine concentration (on a scale of 1–9; see Table 1 for categorical options); and (4) the flavor of e-liquid (tobacco, dessert, fruit, candy, food, beverage, menthol, blended flavors, and other). The categories of concentration and flavor were adapted from the Population Assessment of Tobacco and Health (PATH) study [14]. Participants could choose multiple flavors. Two binary covariates were created for sweet (i.e., dessert, fruit, or candy) and menthol flavors due to their higher prevalence or addictive potential [12].

**Health risk measures.** Participants completed the 37 items of the e-cigarette Wisconsin Inventory of Smoking Dependence Motives (e-WISDM), which was validated using data from dual users of e-cigarettes and combustible cigarettes [15]. The e-WISDM consists of two major scales: the primary dependence motives (PDMs), which measure automaticity, loss of control, craving, and tolerance, and the secondary dependence motives (SDMs), which focus more on instrumental motives: affective enhancement, affiliative attachment, cognitive enhancement, cue exposure/associative processes, social/environmental goads, taste, and weight control. The American Thoracic Society Questionnaire (ATSQ) [16], which has a comprehensive coverage of chronic bronchitis symptoms found in adolescent e-cigarette and cigarette users [17,18], was also administered. Finally, participants were asked about their current use of tobacco products other than e-cigarettes using the categories adopted from the PATH study [14]. A binary variable was created to indicate the current use of any combustible tobacco products, including cigarettes, traditional cigars, cigarillos, filtered cigars, pipes, and hookahs.

### 2.4. Statistical Analysis

To examine the first research objective, a subset of participants who used cartridges, pods, or tanks when they first participated in the study but adopted the new-generation disposables at later waves were identified. Their health risk measures (including e-cigarette dependence symptoms, respiratory symptoms, and combustible tobacco use) and e-cigarette consumption characteristics (including frequency of e-cigarette use, nicotine concentration, and flavor) at the wave before the adoption and at the wave of adoption were compared using paired-sample t-tests (for continuous variables) or McNemar’s tests (for binary variables).

For the second research objective, a generalized linear mixed model (GLMM) with a random intercept (to address the dependence between repeated measures from the same participant) was conducted to investigate the effects of device types on each of the health risk outcomes (primary dependence motives, secondary dependence motives, respiratory symptoms, and combustible tobacco use), adjusting for the effects of other e-cigarette consumption characteristics and demographic background. An identity link was used for the three continuous symptomatology outcomes, whereas a logit link was employed for the binary outcome for combustible tobacco use.

## 3. Results

Table 1 shows descriptive statistics of the demographic background, health risk measures, and e-cigarette consumption characteristics when the 650 participants were first recruited to the study. About half of the participants were male, 70% were White, 17% were Asian, 5% were Black, and 13% were Hispanic, with an average age of 20 and an average amount of money received per week of around USD 101–200. In terms of participant health risk measures, the average primary and secondary e-cigarette dependence scores were both around the middle of the scale (1–7). The average ATSQ score was 1.77 (in the range of 1–5), indicating a low level of respiratory symptomatology. About 35% reported using combustible tobacco in the last 30 days. On average, participants vaped on 25 of the past 30 days, reporting about 10–14 times per day. The mean nicotine concentration category was 40–49 mg/mL or 4.0–4.9%. About 62% participants used a sweet flavor and 69% used a menthol flavor. At baseline, 17% used tanks, 75% used cartridge/pods, and 40% used disposables (participants could use multiple device types). Among the 347 participants with valid device coding in at least two semesters, 26% adopted disposables and 17% adopted tanks during the study period.

Among the 89 participants who started using cartridges, pods, or tanks and later adopted disposables during the 2-year study period, their health risk measures and e-cigarette consumption characteristics reported in the wave before adopting disposables were compared with those reported in the wave when they reported adoption (see the results in Table 2). There was only one statistically significant pre–post difference in health risk measures: the scores of secondary dependence motives were higher after adopting disposables. Regarding e-cigarette consumption characteristics, participants vaped significantly fewer times per day and used higher-nicotine-concentration products after adopting disposables. Furthermore, they were more likely to use sweet flavors but were less likely to use menthol flavors after adopting disposables.

Table 3 delineates the results of the GLMM of the effects of e-cigarette device types on the four health risk measures, controlling for other e-cigarette consumption characteristics and demographic background. Using a tank device was associated with a higher score of primary dependence motives and a higher likelihood to use combustible tobacco. While using a cartridge/pod device was only associated with a higher score of secondary dependence motives, using a disposable was associated with both higher primary dependence motives and higher secondary dependence motives. Some control variables also had significant associations with health risk outcomes. A greater number of days vaping was associated with higher e-cigarette dependence symptomatology but lower odds for combustible tobacco use. A greater number of times vaping per day was associated with higher risks for all four outcomes. Men reported lower e-cigarette dependence and respiratory symptoms but were more likely to use combustible cigarettes. While White e-cigarette users were less likely to use combustible tobacco, Hispanic users had higher odds for combustible tobacco use. E-cigarette users who received a greater amount of money per week tended to have higher e-cigarette dependence.

## 4. Discussion

Using longitudinal data collected during the critical period of the implementation of the FDA’s partial flavor ban and the fast-growing market of disposable e-cigarettes, this study was able to make a unique contribution to the literature by investigating potential effects of adopting new-generation disposables on e-cigarette use behavior and health risks. The results based on the pre–post design among the participants who originally used reusable products show that adopting disposables may increase secondary dependence motives and the use of higher levels of nicotine concentration and sweet flavors but decrease the number of times vaping per day and the use of menthol flavors. These findings are, in general, expected, given that these new-generation products tend to contain higher nicotine concentrations and offer more favorable features to young people such as sweet flavors and a cool appearance [5,6]. The decreased self-reported vaping frequency during the day is a rather unexpected finding because the greater convenience of using disposables compared to cartridges, pods, or tanks [7] may potentially facilitate use behavior. On the other hand, such convenience could also make participants become less conscientious about their actual consumption. In fact, a recent study found that young adult e-cigarette users tend to greatly underestimate their frequency of use according to a comparison between their retrospective reports on a conventional survey question and their real-time reports on EMA [19]. This lack of insight into their actual consumption frequency could increase their risk for e-cigarette dependence and reduce their estimation of the risks posed by their e-cigarette use.

The results based on longitudinal data from this sample of young adult e-cigarette users with various transitions of product use indicate that even after controlling for other e-cigarette consumption characteristics (including use frequency, nicotine concentration and flavors), device types are still related to e-cigarette dependence symptomatology and the likelihood of combustible tobacco use. Tank use is associated with a higher level of e-cigarette dependence that is driven by automatic, heavy use that feels out of control (i.e., PDM) and greater odds of using combustible tobacco. Both of these represent higher health risks, as a higher level of PDM was shown to be associated with heavier daily use and a higher likelihood of relapse [20,21]; using combustible tobacco also exposes people to more toxins related to cancer [22]. Although disposable use is not associated with the likelihood of using combustible tobacco, it is associated with both dimensions of e-cigarette dependence (PDM and SDM), indicating that these new-generation products may increase not only instrumental motives that are influenced by psychological and environmental contexts but also heavy, automatic use that can operate without environmental cues. Moreover, this study shows that it is not the device type, but rather the frequency of vaping, that affects respiratory symptoms. This is consistent with prior findings reviewed in the Introduction [10].

This study has limitations that are important to note. First, the web survey data were self-reported and thus were subject to biases. Second, although the study sites covered both urban and rural settings, the findings are not generalizable to all college student or young adult populations in the US. Third, because the web survey was designed before the FDA’s partial flavor ban, seven popular brands that made cartridges or pods at that time were listed with an open-ended question for participants to input other brand/product names, making the coding of device types challenging. As a result, out of the 1470 records (each participant was measured in multiple semesters), we were not able to generate valid coding for 285 records (19%), which were not included in the analysis. Nevertheless, we adapted an innovative approach using the publicly accessible Google Trends data [1] to conduct the coding, so we were able to limit the amount of missing data.

## 5. Conclusions

In sum, this longitudinal study was able to capture some unintended effects of a public health policy decision. While the intent of the US FDA partial flavor ban was to reduce adolescents’ and young adults’ exposure to e-cigarettes, it is clear that the way the ban was implemented (i.e., permitting flavors in disposable products) allowed people to change their product type. When young adults changed to disposable e-cigarettes, this increased their e-cigarette dependence scores and use of sweet-flavored e-liquids. Several countries (e.g., UK and France) have implemented or have planned to implement bans on all disposable e-cigarettes, citing their adverse health effects and environmental concerns such as hazardous chemicals from discarded batteries and devices. Unlike these countries, the US FDA has not yet completely prohibited the sale of disposable e-cigarettes. Our study findings provided additional evidence for US policymakers to re-evaluate existing tobacco regulatory policies, especially those pertaining to different device types. Although the US FDA has only authorized 23 e-cigarette products, some of which are disposable and only tobacco-flavored e-liquids, adolescents and young adults continue to have access to disposable e-cigarettes in a variety of flavors due to the limited enforcement activity, the prevalence of e-commerce, and illicit importation. At the state level, certain US states (e.g., California, Massachusetts, and New York, USA) have banned any flavored e-cigarettes (except for tobacco flavor), including disposables, but this has not completely refrained vape shops and gas stations from selling them. When these policies are left to individual states, rather than being part of a comprehensive legislative action from the US FDA, state governments often lack the capacity to effectively address the illicit importation of flavored and illegal disposable e-cigarettes, as well as their illegal sale in both retail stores and online shops. It is crucial for the US FDA to continue monitoring new and illicit products and implement more comprehensive tobacco product regulations. This may involve making decisions regarding an entire class of e-cigarettes (e.g., flavored disposable e-cigarettes) rather than individual products.

## Figures and Tables

**Table 1:** Descriptive statistics of the demographic background, health risk measures, and e-cigarette consumption characteristics of the young adult e-cigarette users (N = 650).

	Mean or Count	Standard Deviation or Percent
**Demographic background**		
Male	319	49.08%
White	457	70.31%
Asian	108	16.62%
Black	32	4.92%
Hispanic	86	13.23%
Age	19.83	1.36
Income per week ^a^	2.67	1.67
**Health risk measures**		
Primary dependence motives (PDM) of e-WISDM ^b^	3.78	1.48
Secondary dependence motives (SDM) of e-WISDM ^c^	3.64	1.06
Respiratory symptoms: ATSQ ^d^	1.77	0.66
Combustible tobacco use	225	34.78%
**E-cigarette consumption characteristics**		
Number of days vaping in past 30 days	25.00	7.41
Number of times vaping per day ^e^	2.90	1.53
Nicotine concentration ^f^	7.10	1.80
Sweet flavor	405	62.31%
Menthol flavor	447	68.77%
Tank	109	16.77%
Cartridge/pod	485	74.62%
Disposable	259	39.85%
Device type transition (based on 347 participants with valid device coding in at least two semesters)		
Adopting disposable	89	25.65%
Adopting tank	59	17.00%

^a^ Money received from a job, family, an allowance on a scale of 1 to 12: 1 = USD 0; 2 = USD 1–100; 3 = USD 101–200; 4 = USD 201–300; 5 = USD 301–400; 6 = USD 401–500; 7 = USD 501–600; 8 = USD 601–700; 9 = USD 701–800; 10 = USD 801–900; 11 = USD 901–1000; 12 = USD 1001 or more. ^b^ The PDM of e-cigarette Wisconsin Inventory of Smoking Dependence Motives (e-WISDM) was calculated by averaging the subscales of automaticity, loss of control, craving, and tolerance (on a scale of 1–7). ^c^ The SDM of e-WISDM was calculated by averaging the subscales of affiliative attachment, cognitive enhancement, cue exposure/associative processes, social/environmental goads, taste, weight control, and affective enhancement (on a scale of 1–7). ^d^ The American Thoracic Society Questionnaire (ATSQ) score was the mean of 8 items (on a scale of 1–5). ^e^ On a scale of 1–6: 1 = 0–4; 2 = 5–9; 3 = 10–14; 4 = 15–19; 5 = 20–29; 6 = 30 or more times/day. ^f^ On a scale of 1–9: 1 = 0 (0%); 2 = 1–12 (1.3–1.7%); 3 = 13–17 (1.3–1.7%); 4 = 18–24 (1.8–2.4%); 5 = 25–29 (2.5–2.9%); 6 = 30–39 (3.0–3.9%); 7 = 40–49 (4.0–4.9%); 8 = 50–59 (5.0–5.9%); 9 = 60 mg/mL or more (6.0% or more).

**Table 2:** Pre-post differences among cartridge/pod/tank users who adopted disposables (N = 89).

	Before Adopting Disposables	After Adopting Disposables	Paired *t* Test or McNemar’s Test
	Mean (SD) or Count (%)	Mean (SD) or Count (%)	t$\;\mathrm{or}\;x^{2}$(p-Value)
**Health risk measures**			
Primary dependence motives (PDM) of e-WISDM ^a^	3.89 (1.50)	4.19 (1.53)	1.77 (0.08)
Secondary dependence motives (SDM) of e-WISDM ^b^	3.67 (1.10)	4.01 (1.27)	2.42 (0.02) *
Respiratory symptoms: ATSQ ^c^	1.87 (0.70)	1.75 (0.73)	−1.86 (0.07)
Combustible tobacco use	31 (35.23%)	39 (44.32%)	2.13 (0.14)
**E-cigarette consumption characteristics**			
Number of days vaping in past 30 days	24.27 (8.27)	24.58 (7.67)	0.32 (0.75)
Number of times vaping per day ^d^	2.99 (1.61)	2.65 (1.29)	−2.18 (0.03) *
Nicotine concentration ^e^	6.92 (2.02)	7.51 (1.24)	2.09 (0.04) *
Sweet flavor	51 (57.30%)	77 (86.52%)	22.53 (<0.01) *
Menthol flavor	65 (73.03%)	52 (58.43%)	4.57 (0.03) *

* *p* < 0.05. ^a^ The PDM of e-cigarette Wisconsin Inventory of Smoking Dependence Motives (e-WISDM) was calculated by averaging the subscales of automaticity, loss of control, craving, and tolerance (on a scale of 1–7). ^b^ The SDM of e-WISDM was calculated by averaging the subscales of affiliative attachment, cognitive enhancement, cue exposure/associative processes, social/environmental goads, taste, weight control, and affective enhancement (on a scale of 1–7). ^c^ The American Thoracic Society Questionnaire (ATSQ) on a scale of 1–5. ^d^ On a scale of 1–6: 1 = 0–4; 2 = 5–9; 3 = 10–14; 4 = 15–19; 5 = 20–29; 6 = 30 or more times/day. ^e^ On a scale of 1–9: 1 = 0 (0%); 2 = 1–12 (1.3–1.7%); 3 = 13–17 (1.3–1.7%); 4 = 18–24 (1.8–2.4%); 5 = 25–29 (2.5–2.9%); 6 = 30–39 (3.0–3.9%); 7 = 40–49 (4.0–4.9%); 8 = 50–59 (5.0–5.9%); 9 = 60 mg/mL or more (6.0% or more).

**Table 3:** Generalized linear mixed models of health risk measures on e-cigarette consumption characteristics and demographic background (1185 records).

	Primary Dependence Motives (e-WISDM PDM)	Secondary Dependence Motives (e-WISDM SDM)	Respiratory Symptoms (ATSQ)	Combustible Tobacco Use
Intercept	0.4449 (0.6692)	2.2708 (0.5536)	1.9088 (0.3892)	−0.4688 (1.2242)
**E-cigarette consumption characteristics**				
Tank	0.1998 * (0.0960)	0.1288 (0.0829)	0.0721 (0.0537)	0.4772 * (0.2172)
Cartridge/pod	0.1335 (0.0856)	0.2251 * (0.0739)	−0.0008 (0.0477)	0.3342 (0.1964)
Disposable	0.2158 * (0.0773)	0.2533 * (0.0669)	−0.0645 (0.0429)	0.2514 (0.1793)
Number of days vaping in past 30 days	0.0624 * (0.0049)	0.0358 * (0.0042)	0.0026 (0.0028)	−0.0353 * (0.0108)
Number of times vaping per day ^a^	0.3204 * (0.0248)	0.1568 * (0.0212)	0.0602 * (0.0139)	0.2287 * (0.0548)
Nicotine concentration ^b^	0.0306 (0.0192)	−0.0104 (0.0165)	0.0095 (0.0107)	−0.0229 (0.0421)
Sweet flavor	−0.0673 (0.0796)	0.1150 (0.0685)	−0.0267 (0.0445)	0.0447 (0.1813)
Menthol flavor	0.0370 (0.0761)	−0.0296 (0.0657)	0.0463 (0.0425)	−0.0031 (0.1750)
**Demographic background**				
Male	−0.3526 * (0.0876)	−0.3313 * (0.0723)	−0.2521 * (0.0512)	0.5803 * (0.1577)
White	0.0633 (0.0969)	−0.0480 (0.0799)	−0.0053 (0.0565)	−0.4291 * (0.1708)
Hispanic	−0.1347 (0.1282)	0.0203 (0.1056)	−0.0606 (0.0749)	0.8898 * (0.2197)
Age	0.0263 (0.0319)	−0.0061 (0.0262)	−0.0152 (0.0186)	−0.0359 (0.0568)
Income per week ^c^	0.0667 * (0.0262)	0.0499 * (0.0217)	0.0053 (0.0153)	0.0912 (0.0468)

Note: The numbers in each cell are regression coefficient (standard error). * *p* < 0.05. ^a^ On a scale of 1–6: 1 = 0–4; 2 = 5–9; 3 = 10–14; 4 = 15–19; 5 = 20–29; 6 = 30 or more times/day. ^b^ On a scale of 1–9: 1 = 0 (0%); 2 = 1–12 (1.3–1.7%); 3 = 13–17 (1.3–1.7%); 4 = 18–24 (1.8–2.4%); 5 = 25–29 (2.5–2.9%); 6 = 30–39 (3.0–3.9%); 7 = 40–49 (4.0–4.9%); 8 = 50–59 (5.0–5.9%); 9 = 60 mg/mL or more (6.0% or more). ^c^ Money received from a job, family, an allowance on a scale of 1 to 12: 1 = USD 0; 2 = USD 1-USD 100; 3 = USD 101-USD 200, …, 12 = USD 1001 or more.

## Data Availability

The research team plans to deposit the data through the National Addiction & HIV Data Archive Program (NAHDAP) after the study is concluded, so the data will become publicly accessible.
